# *Mycobacterium smegmatis*, a Promising Vaccine Vector for Preventing TB and Other Diseases: Vaccinomics Insights and Applications

**DOI:** 10.3390/vaccines11081302

**Published:** 2023-07-31

**Authors:** Weile Xie, Longlong Wang, Dan Luo, Vijay Soni, Eric H. Rosenn, Zhe Wang

**Affiliations:** 1 Shanghai Key Laboratory of Veterinary Biotechnology, School of Agriculture and Biology, Shanghai Jiao Tong University, Shanghai 200240, China; 2 Shanghai Collaborative Innovation Center of Agri-Seeds/School of Agriculture and Biology, Shanghai Jiao Tong University, Shanghai 200240, China; 3 Division of Infectious Diseases, Weill Department of Medicine, Weill Cornell Medicine, New York, NY 10065, USA; 4 School of Medicine, Tel Aviv University, Tel Aviv 6997801, Israel

**Keywords:** *M.sm*, vaccine vector, omics, system biology, infectious diseases, vaccinomics, tuberculosis

## Abstract

*Mycobacterium smegmatis* (*M.sm*) is frequently used as an alternative model organism in *Mycobacterium tuberculosis (M.tb*) studies. While containing high sequence homology with *M.tb,* it is considered non-pathogenic in humans. As such it has been used to study *M.tb* and other infections in vivo and more recently been explored for potential therapeutic applications. A body of previous research has highlighted the potential of using genetically modified *M.sm* displaying rapid growth and unique immunostimulatory characteristics as an effective vaccine vector. Novel systems biology techniques can further serve to optimize these delivery constructs. In this article, we review recent advancements in vaccinomics tools that support the efficacy of a *M.sm-*based vaccine vector. Moreover, the integration of systems biology and molecular omics techniques in these pioneering studies heralds a potential accelerated pipeline for the development of next-generation recombinant vaccines against rapidly developing diseases.

## 1. Introduction:

In 1884, Sigmund isolated a novel species of the mycobacterium family which was designated *Mycobacterium smegmatis* (*M.sm*) by Lehmann and Neumann in 1889. *M.sm* is a saprophytic, biofilm-forming bacterium that is non-infectious in mammals. *M.sm* is commonly found in soil, water, and plants endemic to sixteen states, Australia, Russia, Canada, and Switzerland [1]. Its doubling time is almost 3 h, faster than other *Mycobacterium* species, and it grows in non-pigmented, velvety, yellow-colored colonies. The surface morphology of this microbe is classically described as “shiny, smooth, and finely wrinkled or coarsely collapsed” [2]. Although *M.sm* is a strict aerobe, like other *Mycobacterium* species, it may survive through short anaerobic periods during certain stages of its disease course. There are various similarities between *M.sm* and other harmful, infectious *Mycobacterium* species, such as *Mycobacterium tuberculosis (M.tb)*. The most immediately apparent similarity between the two species a common mycothiol biosynthesis pathway for the production of basic thiol, which is required for intra-bacterial homeostasis [3]. The sharing of a key metabolic pathway, even this simple example, eludes to the commonalities between *M.tb* and *M.sm* at multiple functional levels.

Almost twenty years ago, Dr. Trey Ideker defined systems biology as the use of systematic genomic, proteomic, and metabolomic technologies to acquire data for developing models of complex biologic systems and pathologies [4]. By integrating our understanding of how different biologic components function, systems biology aims to enhance our knowledge of living systems and develop predictive models of how they behave when perturbed [5]. Systems biology is, therefore, an interdisciplinary field that includes the studies of biology, computer science, engineering, bioinformatics, physics, and other areas. As opposed to traditional hypothesis-driven research, system biology is a discovery-driven approach employing a new holistic perspective that allows us to see the whole picture of biological systems. It is thus able to elucidate both molecular components and underlying cellular processes. It further predicts how these systems may change over time under different conditions, yielding valuable information for solutions to health and environmental problems [6].

Various techniques and methods based on systems biology have evolved to incorporate cross-disciplinary process analysis systems. These methods are integrative experiment systems that connect multiscale interactions such as molecular, cell, tissue, and organ systems level functions to predict a physiological outcome or organismal phenotypes based on quantitative reasoning, computational models, and high-throughput experimental technologies [7]. A major application of systems biology is represented by systems vaccinology tools developed in the last decade. The use of data sets retrieved from systems-based studies supports the rational design, versus the classic trial-and-error methodology of vaccine development, leading to more effective, safe, and efficacious vaccines [8]. By implementing different techniques (e.g., transcriptomics, proteomics, and cell-based assays) systems vaccinology can be applied to all phases of vaccine development by providing detailed insight into research considerations, such as composition, immunogenicity, and safety of vaccine candidates. With these tools, the potential of an *M.sm* vaccine has gained a new significance [9]. In this review, we discuss the application of systems biology techniques in the development of *M.sm* vector vaccines. We further highlight the potential for future integration of omics technology in improving *M.sm* recombinant vaccines.

## 2. System Biology Understanding of *M.sm*

The complete genome sequence of *M.sm* was made available to the public in November 2006 (https://www.ncbi.nlm.nih.gov/ accessed on 20 November 2006). *M.sm* is a saprophytic, fast-growing strain, although there have been a few cases of human and animal infection reported [10]. This organism yielded early insights that formed the foundation for understanding mycobacterial genetics [11], and has continued to be used as a surrogate host to study the virulence and regulatory pathways of *M.tb* specifically [12,13]. In recent years, comparative genomics has further validated *M.sm* as a useful model for studying the greater *Mycobacterium* genus based on its high homology. With regard to *M.tb* specifically, >2800 orthologs among its ~4000 protein-coding genes were identified in *M.sm.* These furthermore displayed >50% global amino acid identity [14]. The average protein identity between orthologs among various species has been measured as >70% [15]. While this provides a significant genetic basis for comparison, the homology extends even beyond the *M.tb* complex (MTBC). An estimated set of ~1150 core *M.sm* proteins were found to share >50% global amino acid identity with a number of other mycobacterial species including *M. abscessus, M. marinum, M. avium*, and *M. leprae* [14].

These core proteins likely perform similar functional roles in each species, and transposon mutagenesis studies support this 96% of essential genes identified in *M.sm* have homologs in *M.tb*, 90% of which are comparably critical for survival in *M.tb* [16]. The greater genetic organization in each species is also conserved, showing similar patterns of gene co-localization around chromosomes, indicating possible conserved mechanisms of gene regulation. These discoveries have lent support to the theory that many important pathways are, in part, conserved between *M.tb* and *M.sm* [17].

Two conserved elements that are of key importance are termed sigma factors and two-component systems. Sigma factors, a family of extra-cytoplasmic signaling molecules, serve a function in adaptive responses to various environmental stressors and are responsible for some of the virulence traits of *M.tb* [18]. A bioinformatic analysis of the *M.sm* genome has predicted the existence of 26 sigma factors; twice the number that have been identified in *M.tb*. **T**wo-component regulatory systems (2CRSs) are another key player in the bacterial response to changing environmental conditions [19,20]. These systems act by integrating multiple stimuli to effect coordinated changes in global gene expression.

The non-pathogenic MC^2^155 strain of *M.sm* has historically been utilized as a model organism. As such, its transcriptional landscape and genome information data have been extensively mapped and documented, laying a solid foundation for further related research [21]. In transcriptomic studies, the effects of certain genes expressed in *M.sm* have been identified, such as PrrAB-mediated control of many metabolic, respiratory, and dormancy pathways [22]. One commonly used technique to investigate potential gene targets is conducted through overexpression of *M.tb* orthologous genes in *M.sm*. For instance, *Rv2788* overexpression was shown to increase *Mycobacterium* endurance to stress [23]. Finding possible mediators of bacterial survival such as this provides a direction for screening genes with potential implications in vaccine development.

**Figure 1:**
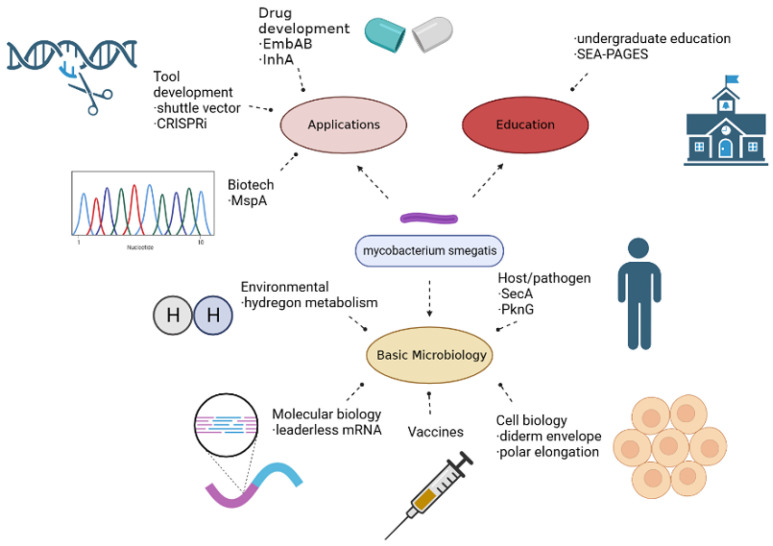
Diagram of *M.sms’* role as a model organism. *M.sm* can make an excellent vaccine vector, and due to its non-pathogenicity can even be tolerated by immunocompromised individuals [26]. *M.sm* as a vector vaccine for HIV/AIDS is therefore of interest, and results of preliminary laboratory work have shown some potential. A pitfall is that the mechanism of vaccine immunogenicity is quite different from *M.tb*, as *M.sm* is unable to enter epithelial cells or survive in phagocytes. The underlying omics basis for such remarkable differences remains to be determined [27]. A few approaches have thus been undertaken to explore how an *M.sm* vaccine promotes immunity despite these differences, and if there are opportunities to take advantage of its unique characteristics.

Over the past decade, the proliferation of technology has allowed us to conduct breakthrough omics research at increasing rates. For example, high-throughput screening of conserved, fluorescently labeled core protein libraries produced in Msr (methionine sulfoxide reductase), yielded unprecedented mechanical data. For example, the relative position of ribosomal proteins which were revealed to be regularly clustered but specifically absent at the nucleoid and polar regions of the cell. As seen here, colocalization of proteins is an important measurable characteristic that could elucidate previously unknown functions or even help identify unclassified proteins. Utilizing *M.sm* as a model organism, this methodology might be extended to gain insight on core protein functional pathways in other organisms [24]. The basis for inferences between the model *M.sm* and *M.tb* is further affirmed by recent genomic advances like CRISPRi gene inhibition technology. Applying this yielded a hallmark study, which through knockdown analysis indicated 263 essential *M.sm* genes with precise orthologues in *M.tb* [25]. Figure 1 shows other uses of *Mycobacterium smegmatis* as a model bacterium.

Based on proteomics studies, *M.sm* antigenicity is most specific for secreted, peptidoglycan-associated protein targets. Cell wall proteome analysis of *M.sm* strain MC2155 shows that type VII secretion (T7S) systems are used by *M.sm* in passing these proteins across the cell envelope [26]. Pathogenic mycobacteria can have five of these secretory systems, commonly named ESX-1 to ESX-5. At least three of these secretory systems are critical to the virulence and viability of *M.tb*. [28,29]. Among the non-pathogenic mycobacteria, like *M.sm,* only ESX-1, ESX-3, and ESX-4 are present [30], Although the specific mechanisms have been well studied [31,32,33], many important ESX-5-related genes in pathogenic bacteria are recombinant and hard to define [34]. As for ESX-2, its role in pathogenic bacteria is still unclear, and no recombination studies have been conducted [35]. Further, elucidating T7S is, therefore, a rational next step in further understanding *M.sm*. Table 1 shows the similarities and differences between *M. smegmatis* and *M. tuberculosis* in ESX gene clusters.

The pathogenesis of mycobacterial infection relies heavily on immune non-dominant virulence factors, secreted by the T7S mechanism, which are involved in all stages of infection, from host colonization to persistence. These days, significant progress has been made in discovering T7S substrates and categorizing the general secretion motif. There are also an increasing number of studies on the expression of recombinant proteins in *M.sm* [36].

## 3. Why Is *M.sm* an Effective Vaccine Vector for Development of Recombinant *Mycobacterium* Vaccines?

### 3.1. Omics-Based Antigen Discovery in Mycobacterium sp.

Searching for *M.tb* antigens that are both recognized by the immune system in *M.tb* infected humans and that provide protective immunity in animal models has been a challenging task. Table 2 describes a number of hallmark studies on advances in this field and compares the homology of the predicted antigens of *M.tb* with that of *M.sm*, most of which were isolated by traditional biochemical methods.

Until recently, most approaches to antigen discovery were based on traditional methods of separating and identifying antigens from complex mycobacterial protein mixtures. While antigens in culture filtrate were initially considered key candidate antigens, the focus has now shifted to protective T-cell antigens found among non-secretory protein isolates. This includes the biochemical identification of the 71-kDa cell wall protein [37,38]. Identification of *M.tb 41* and *M.tb 39a* proteins were also achieved by T-cell expression cloning and serologic expression cloning, respectively [39,40].

Interestingly, sequencing of the *M.tb* genome has revealed that many low-mass antigens belong to common gene families, sharing genetic organization and thus similar antigenicity/immunogenicity. Due to difficulty with isolating specific low-mass antigens in significant amounts, this genetic basis has been considered for the isolation of functional target groups instead. The primary biochemical approach for target molecule identification has thus been replaced by direct T-cell antigen screening techniques based on genomic analysis.

**Figure 2:**
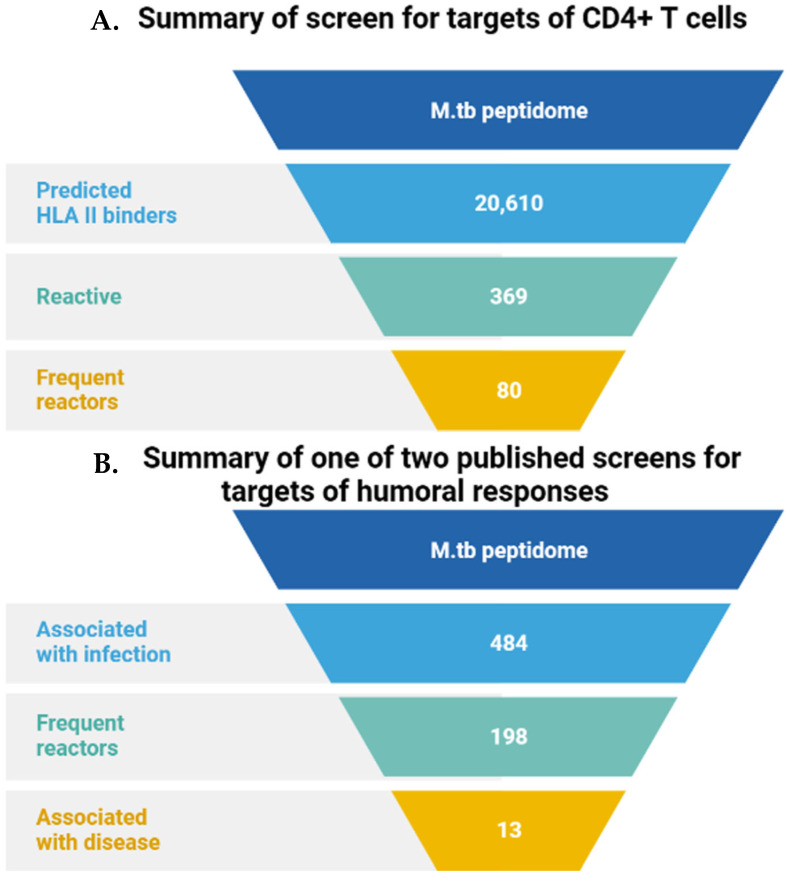
Summary of immunoantigen analysis of *M.tb* based on proteomics. (**A**) CD4+ T cell targets—Sequences representing 5 intact and 16 incomplete *M.tb* genomes were screened by HLA Class II consensus method, predicting binding activity to 22 commonly expressed HLA-DR, -DP, and -DQ alleles. The high-affinity peptide was synthesized and the stimulation of IFN-γ produced by circulating T cells of 28 latently infected unvaccinated BCG donors from non-endemic areas was detected by ELISPOT. Of the 369 active peptides, 80 peptides accounted for ~75% of the reactions [42]. (**B**) Humoral response target screening—Proteins related to *M.tb* infection were characterized by comparing the responses between TB and non-TB patient groups [43]. A total of ~95% of *M.tb* open reading frame (H37Rv strain) corresponding to 3988 proteins was cloned into an *Escherichia coli* expression system. Unpurified protein product was printed directly onto nitrocellulose-coated slides, then probed with serum samples from uninfected healthy individuals from a non-endemic region (*n*  =  64) and from suspected TB carriers from endemic regions (*n*  =  561). The reaction of a protein with endemic region samples but not uninfected individuals’ samples identified an antigen. A total of 198 of 484 antigens reacting with multiple endemic region samples were termed ‘frequent reactors’.

Modern bioinformatics techniques of whole genome analysis e can quickly group genes into different families (for example, the ESAT-6 family). Combined with the recognition of specific coding sequences, this allows for precise searches of other candidate molecules within the same gene family. With the development of high-throughput peptide and protein synthesis techniques it is, therefore, now possible to thoroughly analyze the entire *M.tb* proteome to look for “hit” antigens. “Lead” CD4+T cells are commonly used in these proteomic approaches [41] (Figure 2).

Computer algorithms developed to search for peptides that have a high binding affinity with human MHC molecules can either be applied to the whole genome [44,45] or to a more limited number of genes. These might include specific targets initially identified by conventional purification or by one of the methods described above [46]. With the help of these tools, the whole proteome of *M.tb*, or subsets of specific interest can be analyzed in silico for T cell epitopes. The resulting peptides can then be considered for evaluation in vitro based on their ability to stimulate T-cells. This approach has proven effective in the study of various other pathogens, generating a range of targets [47], and more limited data support this methodology for *M.tb* as well [48].

### 3.2. Preliminary Application of M.sm Vector Vaccine

The development of omics has helped us to understand more about *mycobacteria* sp. based on the homology between *M.sm* and *M.tb*. Omics research has further revealed the advantages of *M.sm* as a model strain with important applications in vector vaccine research.

**Figure 3:**
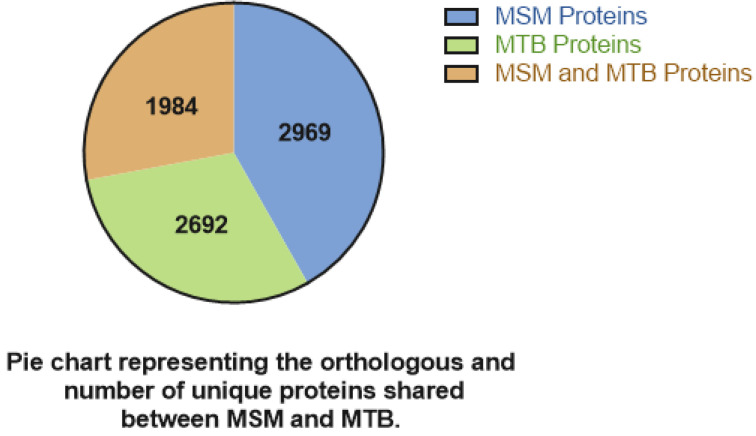
Proteins shared between *M.sm* and *M.tb* genome. The blue part is a protein specific to *M. smegmatis*, the green part is a protein specific to *M. tuberculosis*, and the orange part is a protein shared by both.

As *M.tb* antigen screening proved effective and significant coverage was established, research interest expanded to the construction of vaccines based on *M.sm*. Early experimental vaccines based on the cell wall components of *M.sm* triggered cross-reactivity against *M.tb* antigens in mice [49,50,51]. Figure 3 shows the protein homology of *M. smegmatis* and *M. tuberculosis*. Due to the evident sequence homology, *M.sm* came under consideration as an effective vaccine vector for *M.tb*.

In the study of *M.tb*, the three virulence-related gene clusters ESX-1, ESX-3, and ESX-5 were mainly studied as antigen targets first. After it was found that the cell wall of *M.sm* can induce an immune response, whether to express the associated antigens of *M.tb* on its surface has become a new research hotspot.

Table 3 summarizes the research and application of *Mycobacterium smegmatis* vector vaccine in anti-tuberculosis.

In a basic early study, G. Harth et al. performed heterologous expression and secretion construction of four major *M.tb* extracellular proteins (30-, 32-, 16-, and 23.5 kDa proteins—the first, second, third, and eighth most abundant proteins, respectively) in a rapidly growing non-pathogenic *Mycobacterium* species. This was the first time that a recombinant *M.tb* extracellular protein had been secreted in its native form [52]. Other early studies looked at the ESX-3 gene and showed a set of mycobacterial genes in the ESX-3 region directly linked to the targeting of *M.tb* by the innate immune response.

Miao Xu et al. found *M.sm* vaccine derived from *M.sm* showed strong immunogenicity, promoted Th1 responses, and inhibited the Th2 response in mice [53]. *Sweeney* et al. investigated the role of ESX-3 loci in the pathogenesis of mycobacteria. The study uncovered a previously unrecognized function of ESX-3 loci in innate immune evasion. They further found that *M.sm* with the deletion of the ESX-3 gene could serve as a new vaccine vector with enhanced innate immune activation properties. When engineered to express *M.tb* ESX-3, the vector was found to be an effective TB vaccine that was able to provide a level of protection superior to BCG when administered intravenously [54]. Thus, recombinant *M.sm* can exert immunotherapy effects in *M.tb* infections.

The ESX-1 and ESX-5 genes have also been thoroughly studied to classify their role in *Mycobacterium* virulence; however, the ESX-5 gene in particular is absent in *M.sm.* Here, we see a more recent example of small differences between *M.tb* and *M.sm* that can be leveraged for insight. By serving as a surrogate host for *M.tb* viral genes, the normally non-pathogenic *M.sm* can help elucidate their pathogenic and immunomodulatory mechanisms. For instance, the PE/PPE protein family was revealed via recombination studies to be encoded by *esx-5* genes and is likewise an essential component of the bacterial cell wall, indicating likely antigenicity and bringing it into focus as a potential viral vector. Kaisar Ali et al. explored this hypothesis by testing the PE subfamily member PE31 (*Rv3477*) function in virulence and host–pathogen interactions. By expressing the *M.tb* PE31 in the non-pathogenic *M.sm* strain (Ms_PE31), they isolated the interaction of this protein with the host immune system; any pathogenic response observed would be due to the presence of the protein. PE31 was thus identified as a functionally relevant virulence factor of *M.tb* and as a potential vaccine target for ESX-5 [55]. Operating on this principal Wenmin Yang et al. successfully recombined PE_PGRS18 in *M.sm*. The addition of the protein was found to alter the production of host cytokines IL-6, IL-1β, IL-12p40, and IL-10, and also enhanced survival within macrophages by halting apoptosis [56]. Guoying Deng et al. similarly investigated the potential role of *Rv0431*, a membrane protein associated with glycosylation, finding it to be associated with mycobacterial virulence [57]. T. Garbe et al. created a recombinant 19 Dalton glycosylation-associated protein in *M.sm*. The recombinant strain effectively stimulated the proliferation and differentiation of T cells [58]. Shanshan Sha et al. generated a recombinant *M.sm* expressing *M.tb* Rv1987, encoded by the region of difference (RD)-2 gene. Even though the region of difference (RD)-2 gene is not a virulence-related gene, like *esx-5*, the recombinant strain still effectively stimulated the proliferation and differentiation of T cells [59]. These results suggest that the PE/PPE protein family and analogous cell wall proteins are excellent targets for recombinant vaccine design.

Many of the proteins secreted by ESX-1 are immunodominant and, therefore, at the forefront of infection and disease research. The major secreted proteins of ESX-1 are antigenic target 6-kDa antigen (ESAT6) and culture filtrate protein 10 (CFP10). These essential heterodimeric complexes have become major candidates of recombinant studies, and a number of representative achievements in advancing the understanding of ESX-1 in this regard are listed below. From the recombinant construction of single antigens to the combined expression construction of multi-antigens, the evaluation of immune complexes has acquired the more multi-dimensional nature of a systems biology aproach. Yan Li et al. separately studied a recombinant *M.sm* expressing *M.tb* ESAT-6 gene and found the strain had strong immunogenicity [60]. H. Zhang et al. discovered a *M.sm-*based vaccine expressing an ESAT6 and CFP10 fusion protein. The protective efficacy of the vaccine rM.S-e6c10 was found to be similar to that of the BCG vaccination, based on measurements of *M.tb* burden and lung pathology. The result suggests that the recombinant *M.sm* vaccine expressing the ESAT6-CFP10 fusion protein shows the potential for clinical application [61].

In addition to the three crucial *M.tb* virulence genes mentioned above, *M.tb* has a series of genes that encode important antigenic proteins, such as the Ag85 complex(including Ag85A, Ag85B, Ag85C) and MPT64.

Nur-Ayuni Kadir et al. evaluated the immunogenicity of *M.sm* expressing three T cell epitopes from *M.tb* Ag85B (P21, P26, and P53) in a murine model. The results showed that the resistance of total immunoglobulin G and its subclasses to Ag85B epitopes in the serum of immunized mice was significantly increased [62]. Devin R. Lindsey et al. subsequently proposed the idea of constructing an *M.sm* vaccine design that overexpresses Ag85B for treating immunodeficient patients. After successfully constructing the recombinant strain, they compared its immunogenicity with that of the wild type. The results confirmed that the recombinant strain could effectively stimulate and increase the number of CD4+ IFN-γ + T cells in the lung [63].

*M.tb* has been shown to release MPT64 (*Rv1980c*) protein in high amounts in patients with active tuberculosis. P. W. Roche et al. constructed a recombinant *M.sm* strain expressing MPT64 protein that was found to stimulate human rMPB64 T cell lines, establishing the T cell reactivity of the MPT64 protein. This result suggests that the recombinant strain has the potential to be used as a skin reagent for tuberculosis infection [64]. Nisha Kannan et al. further studied this interaction, generating an *M.sm* strain expressing MPT64 protein by recombination with an *M. avium*-derived antigen gene rather than that of *M.tb*. Testing revealed immunization using above recombinant vaccine can confer equal protection against *M.avium* infection as the *Mycobacterium bovis* BCG, and strongly induced IL-17 to stimulate CD4+ and CD8+ T cell responses [65].

In studying the recombinant expression of single antigen genes, BCG is an excellent cloning candidate due to its homology with *M.sm*. Valeria Falcone et al. used the genomic library of BCG to generate multiple recombinant *M.sm* strains. The strains produced showed a high splenic survival rate compared to attenuated *M.tb*, which further supports the use of *M.sm* for recombinant vaccine development [66]. In addition, Manaswini Jagadeb et al. used an immunoinformatic approach to identify potential peptide-based TB vaccine targets in response to BCG’s limitations. In the modeled structures of selected proteins, T-cell and B-cell epitopes, as well as MHC molecular binding efficiency, were discovered and mapped. Two peptides, Pep-9 and Pep-15, were next discovered through the calculation of antigenicity scores and molecular dynamics simulations in conjunction with MHC-I and MHC-II structures. The results indicated that both peptides were non-cytotoxic and could induce the secretion of pro-inflammatory cytokines in stimulated macrophages [67]. This new avenue for rational design has streamlined the identification of powerful antigen subunits which, if successfully expressed in *M.sm* using recombinant techniques, would produce a potent vaccine construct.

Another achievement was made with the production of recombinant strains incorporating multiple genes, effectively producing multiple antigenic proteins. Anthony G. et al. first successfully expressed *M.tb* antigen 85B and ESAT-6 as recombinant fusion proteins in *M.sm*. This study serves as further proof that vaccine candidates from *M.tb* can be expressed in a surrogate mycobacterial host [68]. Wang Ping et al. constructed a recombinant *M.sm* (rMS) expressing Ag85B and ESAT6 fusion protein (AE-rMS). After antigen fusion expression, AE-rMS immunized C57BL/6 mice with strong stimulation of IFN-γ and IL-2, which produced mainly th1 type immune response. Specifically, this leads to robust production of spleen cells and increases antigen-specific cytotoxic T lymphocytes (CTL) activity [69]. In further research, Xiao-Qing Guo et al. constructed a *M.sm* recombinant strain expressing a fusion protein (rMS-Hsp65/IL-2) that is composed of heat shock protein 65 (Hsp65) and human interleukin 2 (IL-2), and next explored the effect of this construct on lymphocyte function in mice. The rMS-Hsp65/IL-2 markedly enhances lymphocyte function. They considered the fusion protein generated by rMS-Hsp65/IL-2 to be of potential value in developing an effective tuberculosis vaccine [70]. Shanmin Zhao et al. constructed a recombinant strain of *M.sm* (rMS) to express the fusion protein of heparin-binding hemagglutinin (HBHA) and human interleukin-12 (il-12). The immune response induced by rMS in mice and its protective effect against *M.tb* were studied. The results showed demonstrated that compared with BCG vaccination, the novel rMS enhanced the th1 cell response (IFN-γ and IL-2) in mice and reduced the bacterial load in the lungs [71].

C. Yang et al. investigated the effect of recombinant *M.sm* (rMS) harboring a co-expression plasmid-encoding human granulysin (GLS) and mouse interleukin-12 (IL-12) on *M.tb* infection in mice. It was found to have an immunogenic effect by stimulation of Th1 response and GLS antimicrobial activity [72]. On this basis, Zhengjun Yi et al. constructed a viable therapeutic vaccine involving IL-12/GLS (granulysin) gene transfer mediated by recombinant *M.sm*. When introduced to macrophages in a BALB/c murine model, the vaccine was found to strongly induce specific Th1 responses against *M.tb*, with no observed side effects [73]. In another study, Ana Paula Junqueira-Kipnis et al. generated an *M.sm* recombinant strain with partial sequences containing *M.tb* genes Ag85c, MPT51, and HspX (CMX). A vaccine formulated with the recombinant strain induced significant immune responses in mice, and conferred greater protection compared to wild-type *M.sm* and BCG-based vaccine treatment groups [74].

These are some of the initial applications of *M.sm* vector vaccines, and although most of them are related to the development of anti-TB vaccines, the result has broad implications for vaccinomics. Based on the current success, the potential *M.sm* vector vaccine is becoming a more extensively researched direction.

## 4. Broad Spectrum Applications of *M.sm* Vector Vaccines

Because of its excellent antigenicity as a vaccine carrier, *M.sm* has the potential to be used in vaccine development not only against *M.tb*, but also other pathogens. As mentioned earlier, neoantigen epitopes for many viruses have been discovered in recent years using computational omics approaches. This technology has allowed for rapid candidate vaccine antigen identification and accelerated the design of recombinant vaccines with a broad range of applications in many pathologies. Table 4 summarizes the research and application of *Mycobacterium smegmatis* vector vaccines in the prevention of other diseases.

*Mycobacterium smegmatis* vector vaccine is a representative application of *Helicobacter pylori* and *Escherichia coli* in the prevention of other bacterial diseases. Lin Lü et al evaluated the ability of *H. pylori* outer membrane protein 26kda antigen (Omp26) to induce therapeutic protection against *H. pylori* infection in mice. Omp26 was cloned and expressed in *M. smegmatis*, fusing with *M. fowleri* β-lactamase protein. They found that immunizing *Helicobacter pylori* infected mice with ms-omp26, by oral administration, significantly reduced bacterial colonization in the stomach [75,76]. Vasconcellos et al. studied a recombinant *Mycobacterium smegmatis* constructed to ex-press BfpA or intimin from enteropathogenic *E. coli* (EPEC). It was found that TNF-α and INF-γ were produced in vitro by splenocytes from mice immunized with recombi-nant BfpA, whereas only TNF-α was produced recombinant endomembrane immun-ized controls. Adhesion of EPEC (E2348/69) to HEp-2 target cells was blocked by IgA or IgG antibodies produced from mice immunized with recombinant BfpA or intimin [77].

*M.sm* recombinant vaccines have many applications in the prevention and treatment of viral diseases, such as hepatitis B and AIDS. Qiaohong Yue et al. generated a recombinant *M.sm* strain expressing the CS1 antigen, a fusion protein consisting of the HBV truncated core protein (amino acids 1–155), and the PreS1 peptide (amino acids 1–55). Compared with DNA vaccines, they found that vaccination with the recombinant strain induced a more robust cellular immune response with a longer duration of humoral immune response [78]. In another study, Ciaran Skerry et al. generated recombinant *M.sm* strains overexpressing genes encoding various *Mycobacterium bovis BCG* lipoproteins to determine the role of TLR2-stimulated lipoproteins on mycobacterial mediated HIV infection in CD4 + T cells [79]. In a subsequent study, Mark J Cayabyab et al. generated a recombinant *M.sm* strain expressing the entire HIV-1 HXBc2 g120 envelope protein. In testing the strains’ immunogenicity, they found it could induce both effector and memory T lymphocytes. Furthermore, after repeated immunization, a stable virus-specific central memory pool was generated [80].

On this basis, Jae-Sung Yu et al. generated and tested another set of recombinant *M.sm* vectors that expressed the HIV-1 group M consensus envelope protein (Env) as a surface, intracellular, or secreted protein. Although the vaccine does not induce an anti-HIV-1 envelope antibody response due to insufficient expression of the inserted protein, they suggested that recombinant *M.sm* immunization could initiate HIV-1 Env protein expression and induce an associated immune response by further improvement is needed to promote the induction of anti-HIV-1 antibody response. [81]. Byoung-Jun Kim et al. has developed a novel pMyong2-based shuttle vector system designed for recombinant *M.sm*. Analysis of its cellular and humoral immune responses against HIV gag proteins showed it elicited a more potent immune effect, determined by higher levels of HIV-1 Gag-specific CD4 and CD8 T lymphocyte proliferation, INF-γ ELISPOT cell induction, and antibody production by comparing with the recombinant *M.sm* using the pAL and pMV306 plasmids [82].

Recombinant *M.sm* also has promising applications in some autoimmune diseases. Ling Chen et al. constructed a recombinant *M.sm* containing the fusion gene Ag85a-IL-17a, which expressed the fusion protein Ag85A-IL-17A displaying effective immunogenicity and that induced the production of specific IL-17A autoantibodies in mice [83]. Next, a mouse model of neutrophil asthma was used to further investigate the effect of rMS on airway inflammation and findings showed recombinant vaccine-induced production of interleukin-17A autoantibodies significantly reduced airway inflammation [84]. Chiara Nicolò et al. infected SJL mice with a *M.sm* based vaccine expressing a chimeric protein that comprises the self-epitopes of proteolipid protein 139–151 (p139) and MPT64 to study autoimmune encephalomyelitis. They found a significant reduction in disease severity compared to immunization with p139 alone [85]. Vrushali Deshpande et al. developed an *M.sm* vector to secrete recombinant tripeptide constructs (AHC; peptides from Apolipoprotein B, Heat-shock protein 60, and *Chlamydia pneumoniae* outer membrane protein) and found that when administered orally, it induced regulatory immune responses and reduced the development of atherosclerosis in a mouse model. The effect was comparable to the potency of common therapies delivered by purified AHC protein [86].

In recent years, *M.sm* has gradually emerged as a form of cancer vaccine. J. L. Haley et al. have successfully engineered *M.sm* to express and secrete biologically active human tumor necrosis factor-alpha (TNF-α). They found that the transgenic *Mycobacterium* was more effective than wild-type controls in inducing or upregulating a range of anticancer cytokines [87]. Sarah L Young et al. similarly performed studies with TNF-α-secreting recombinant *M.sm* and further demonstrated that the expression of mammalian cytokines is a significant basis for the antitumor properties of recombinant *M.sm* vaccines [88]. To generate novel anti-tumor DNA vaccines, MAGEA3 and SSX2 expression vectors were constructed in *M.sm* recombinant strains by Wen Jian et al. and found to have significant antitumor activity [89]. Hyein Jeong et al. introduced a recombinant *M.sm* (rsmeg-hif-hil-7) vaccine that delivers fusion proteins of human macrophage migration inhibitor (MIF) and IL-7, which serve as target antigens and cancer vaccine adjuvants, respectively. Their data showed that rsmeg-hif-hil-7 exhibited a solid anti-tumor immune response in mouse models. They also found that the anti-cancer effects associated with immune checkpoint inhibitors were enhanced. These results suggest that rSmeg-hMIF-hIL-7 is a promising adjuvant for cancer immunotherapy [90].

## 5. Conclusions

Balancing efficacy and cost is a major issue in achieving widespread distribution of vaccines to combat disease in developing countries. Because of differences compared to developed countries, the very limited annual health budgets of many developing countries may hinder or delay the use of advanced and expensive vaccine technologies (such as primary/booster or multicomponent subunit vaccines), even if they are highly effective. As a result, we are faced with the paradox of the infectious disease market—a potentially huge market with a clear public health need, but a high-risk venture in terms of recouping the cost of vaccine development. Thus, getting new vaccines through clinical trials requires not only expanding research efforts, but also reasonably limiting research to ideal antigens and delivery systems with a higher likelihood of producing effective therapies.

**Figure 4:**
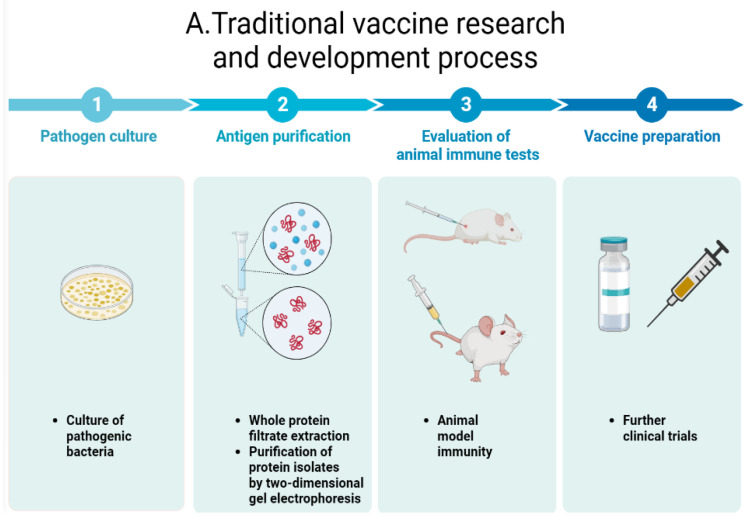

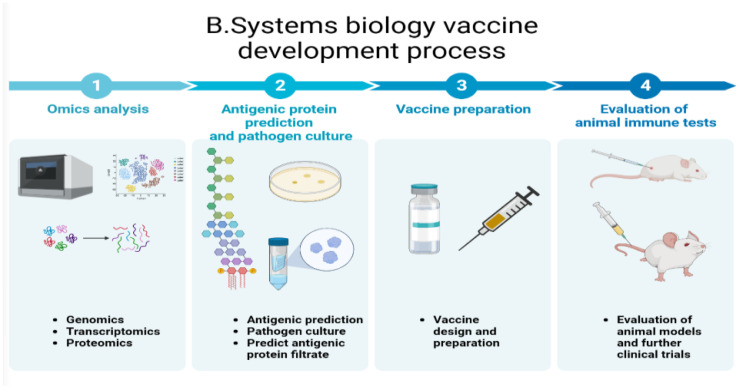
Vaccine research and development process. (**A**) Traditional vaccine research and development process. (**B**) Systems biology vaccine development process. Traditional vaccine development is mainly based on biochemical methods to purify the protein expression of pathogenic bacteria, and then evaluate the feasibility of the protein as the basis of vaccine antigen according to the immunogenicity of the protein, which has a long-time cycle and low efficiency. Omics vaccine development is mainly based on high-throughput omics analysis to select antigen candidates, through the combination of multi-omics analysis to make antigen identification more accurate and more efficient.

Figure 4 summarizes the similarities and differences between traditional vaccine design and omics perspectives on vaccine development.

*M.sm* has been at the forefront of *Mycobacterium* research, not only revealing new strategies for addressing mycobacterial diseases but also providing unexpected insights and valuable approaches where other model bacteria and delivery systems have proved lacking. Because of its high homology and antigenicity with *M.tb*, it can be used as a new vaccine design vector. Defining the repertoire of antigenic targets is central to understanding the immune response against this pathogen. Due to limitations in information collection, data from clinical trials of *M.sm* vector vaccines are not mentioned above. We speculate that this information may not have been yet made publicly available due to the confidentiality of these studies.

In recent years, various omics methods based on systems biology have revealed high-titer antigens of some pathogens, which can be used as foreign genes for recombinant expression in *M.sm* to design vaccines. Advances in proteome-wide screening methods now enable a more extensive and unbiased survey of antigenic targets for complex pathogens like *M.tb*. Some autoimmune diseases and cancers can also be treated via immunization with *M.sm* modified with related cytokines. The progress made in improving the development of *M.sm* vaccines is highly encouraging. In this light, novel licensed recombinant *M.sm* vaccines for preventive and therapeutic use in humans and animals may become a reality in the near future.

## Figures and Tables

**Table 1:** The different ESX systems found in *M.tb* and *M.sm*. The table shows the gene cluster composition of *M. smegmatis* and *M. tuberculosis* in the Type VII secretory system, with circles representing gene clusters shared by both and triangles representing gene clusters specific to *M. tuberculosis*.

	Species	ESX-1	ESX-2	ESX-3	ESX-4	ESX-5	Pathogenicity
RGM	*M.smegmatis*	●		●	●		saprophyte
SGM	*M.tuberculosis*	●	▲	●	●	▲	pathogen

**Table 2:** Identification of antigenic proteins in *M.tb* that are currently known in *M.sm*.

Antigen	Sanger ID	Methods of Identification	Present in CF	Theoretical Mass (kDa)	PATRIC Annotation	Homologous Genes Sanger ID in *M.sm* (Identity)
*ESAT-6*	*Rv3875*	Biochemical purification	+	9.9	Early secretory antigenic target EsxA	*MSMEG0066* (71.56%)
*Ag85A*	*Rv3804c*	Biochemical purification	+	35.7	Secreted antigen 85-A FbpA	*MSMEG2078* (69.21%)
*Ag85B*	*Rv1886c*	Biochemical purification	+	34.6	Secreted antigen 85-B FbpB	*MSMEG2078* (71.08%)
*MPT51*	*Rv3803c*	Biochemical purification	+	28.7	Secreted MPT51/MPB51 antigen protein FbpD	*MSMEG6396* (67.00%)
*MPT64*	*Rv1980c*	Biochemical purification	+	24.8	Immunogenic protein	*MSMEG2331, MSMEG1051* (42.73%)
*CFP10 a*	*Rv3874*	Molecular cloning serological expression cloning	+	10.8	ESAT-6-like protein EsxB	*MSMEG0065* (61.00%)
*TB10.4 a*	*Rv0288*	Antibody expression cloning	+	10.4	Antigen 7 EsxH	*MSMEG0621* (75.79%)
*M.tb8.4*	*Rv1174c*	Biochemical purification	+	10.9	T cell antigen	*MSMEG4804* (52.29%)
*HspX*	*Rv2031c*	Biochemical purification	–	15.4	Heat shock protein	*MSMEG3932* (60.84%)
*CFP6*	*Rv3004*	Biochemical purification	+	12.2	Antigen 6	*MSMEG2371* (60.71%)
*M.tb12*	*Rv2376c*	Biochemical purification	+	12.5	Antigen CFP2	*MSMEG3903* (38.81%)
*M.tb9.9 a antigens*	*Rv1793, MT3721, Rv1198, Rv1037c, Rv3619c*	T cell expression cloning	+	9.8–9.9	ESAT-6 like protein	/
*M.tb32A*	*Rv0125*	Antibody expression cloning	+	32	Serine protease	*MSMEG6289* (49.83%)
*PstS-1*	*Rv0934*	Affinity purification	+	38.2	Phosphate uptake surface protein	*pstS* (34.23%)
*PstS-2*	*Rv0932c*	Antibody expression cloning	+	37.8	Phosphate uptake surface protein	*pstS* (48.47%)
*PstS-3*	*Rv0928*	Antibody expression cloning	+	37.9	Phosphate uptake surface protein	*pstS* (48.42%)
*MPT63*	*Rv1926c*	Biochemical purification	+	16.5	Immunogenic protein	*MSMEG5412* (54.40%)
*M.tb39*	*Rv1196*	Serological expression cloning	–	39.1	PPE family protein	*MSMEG0619* (41.18%)
*M.tb41*	*Rv0915c*	T cell expression cloning	–	41.4	PPE family protein	*MSMEG0619* (42.00%)
*MPT83*	*Rv2873*	Antibody expression cloning	–	22.1	Cell surface lipoprotein	*MSMEG5196* (40.37%)
*71-kDa*	*Unknown*	Biochemical purification	–	71	Unknown surface protein	/
*PPE 68*	*Rv3873*	Comparative genomics	–	37.3	PPE family protein	*MSMEG0064* (62.57%)
*LppX*	*Rv2945c*	Comparative genomics	+	24	lipoprotein	/
	*Rv3878*	Comparative genomics	?		ESX-1 secretion-associated protein EspJ	/
	*Rv3407*	In silico prediction	?	10	Antitoxin	/
	*Rv1818c*	Molecular cloning	–	40.7	PE-PGRS family protein	/

**Table 3:** Recombinant mycobacteriosis vaccines based on *M.sm.*

	Main Author	Antigen	Results/Influences	Reference
Mycobacteriosis	G. Harth et al., 1997.	*M.tb* four major extracellular proteins (the 30-, 32-, 16-, and 23.5-kDa proteins)	Extracellular protein recombination of *M.tb* first achieved	[52]
	Miao Xu et al., 2005.	*M.sm* autologous vaccine	Strong immunogenicity promoted Th1 responses and inhibited Th2 response in mice	[53]
	Sweeney et al., 2011	*M.tb* Esx-3	Superior protection to BCG observed (intravenous administration trial)	[54]
	Md Kaisar Ali et al., 2020	*M.tb* PE subfamily member PE31 (*Rv3477*)	PE31 identified as a potential vaccine target for ESX-5	[55]
	Wenmin Yang et al., 2017	*M.tb* PE_PGRS18	Increased host interleukin production and survival in infected macrophages	[56]
	Guoying Deng et al., 2016	*M.tb Rv0431*	Implications in mycobacterial immunity	[57]
	T. Garbe et al., 1993	*M.tb* 19 Dalton glycosylation-associated protein	Stimulation of T cell proliferation and differentiation	[58]
	Shanshan Sha et al., 2017	*M.tb Rv1987* encoded by the region of difference (RD)-2 gene	Stimulation of T cell proliferation and differentiation	[59]
	Yan Li et al., 2006	*M.tb* ESAT-6 protein	Greater immunogenicity achieved	[60]
	H. Zhang et al., 2010	*M.tb* ESAT-6 and CFP10 protein	Protective efficacy was found similar to BCG	[61]
	Ayuni Kadir et al., 2016	*M.tb* Ag85B	Resistance of serum IgG and its subclasses to Ag85B epitopes significantly enhanced in immunized mice	[62]
	Devin R. Lindsey et al., 2009	*M.tb* Ag85B(overexpressed)	Effective stimulation and increased numbers of CD4+ IFN-γ + T cells observed in lung	[63]
	P. W. Roche et al., 1996	*M.tb* MPT64	Recombinant vaccine’s T cell reactivity is demonstrated by the ability to stimulate human rMPB64 T cell line	[64]
	Nisha Kannan et al., 2020	*M. avium* MPT64	BCG-like protection was observed with strong induction of IL-17; producing CD4+ and CD8+ T cells	[65]
	*Valeria Falcone et al., 1995*	Genomic library of BCG	Superior splenic survival rate observed	[66]
	Manaswini Jagadeb et al., 2021	*M.tb* Pep-9 and Pep-15	Secretion of pro-inflammatory cytokines induced in stimulated macrophages	[67]
	Anthony G. et al., 2013	*M.tb* Ag85B and ESAT-6	Proof for effective expression of major antigens (with dimer variation) secreted by *M.tb*	[68]
	Ping Wang et al., 2014	*M.tb* Ag85B and ESAT-6	Strong stimulation of spleen cells producing IFN-γ- and il-2 and increased activity of antigen-specific cytotoxic T lymphocytes (CTL) to produce TH1 type immune response	[69]
	Xiao-Qing Guo et al., 2012	A fusion protein of heat shock protein 65 (Hsp65) and human interleukin 2 (IL-2)	Lymphocyte function markedly enhanced	[70]
	Shanmin Zhao et al., 2012	A fusion protein of heparin-binding hemagglutinin (HBHA) and human interleukin 12	Enhanced Th1-type cellular responses (IFN-γ and IL-2) in mice and reduced bacterial burden in lungs compared to BCG vaccination	[71]
	C. Yang et al., 2009	A co-expression plasmid encoding human granulysin (GLS) and mouse interleukin-12 (IL-12)	Immunotherapeutic effect found to be related to stimulation of Th1 response and GLS antimicrobial activity	[72]
	Zhengjun Yi et al., 2007	IL-12/GLS (granulolysin)	Strong induction of specific Th1 responses against *M.tb*	[73]
	Ana Paula Junqueira-Kipnis et al., 2013	*M.tb* Ag85c, MPT51, and HspX	Significant immune responses induced	[74]

**Table 4:** Recombinant vaccines for other diseases based on *M.sm*.

	Main Author	Antigen	Results/Influences	References
Gastrointestinal Diseases	Lin Lü et al., 2009, 2011	*Helicobacter pylori* (*H. pylori*) outer membrane protein 26kDa antigen (Omp26)	Inducing protection and a significant reduction in bacterial colonization in the stomach	[75,76]
	Vasconcellos et al., 2012	(enteropathgenic) *E. coli* BfpA or intimin	Effectively stimulate the production of TNF-α and INF-γ	[77]
Viral Diseases	Qiaohong Yue et al., 2007	A fusion protein consisting of the HBV truncated core protein (amino acids 1–155) and the preS1 peptide (amino acids 1–55)	A stronger cellular immune response and a longer duration of humoral immune response were induced	[78]
	Ciaran Skerry et al., 2016	*Mycobacterium bovis* BCG lipoproteins	Determining the role of potential TLR2-stimulated lipoproteins on mycobacterial mediated HIV infection in CD4 + T cells	[79]
	Mark J Cayabyab et al., 2006	HIV-1 HXBc2 gp120 envelope protein	Inducing both effector and memory T lymphocytes and generating a stable virus-specific central memory pool	[80]
	Jae-Sung Yu et al., 2006	HIV-1 group M consensus envelope protein	Insufficient expression of insertional protein can still effectively generate immune response against HIV	[81]
	Byoung-Jun Kim et al., 2017	HIV gag proteins	Eliciting more potent immunity	[82]
Autoimmune Diseases	Ling Chen et al., 2015	A fusion protein Ag85a-IL-17A	Inducing the production of interleukin-17A autoantibodies that reduced airway inflammation in mice with neutrophil asthma	[83]
	Wanting Xu et al., 2022	A fusion protein Ag85A-IL-17A	Obtaining in the immune test of asthmatic mouse model established by ovalbumin	[84]
	Chiara Nicolò et al., 2010	A chimeric protein containing the self-epitope of proteolipid protein 139–151 (p139) fused to MPT64	The disease severity was significantly reduced by comparing with p139 alone	[85]
	Vrushali Deshpande et al., 2016	Tripeptide constructs (AHC; peptides from Apolipoprotein B, Heat-shock protein 60 and *Chlamydia pneumoniae* outer membrane protein)	Inducing regulatory immune responses and reducing the development of atherosclerosis in a mouse mode	[86]
Cancer	J. L. Haley et al., 1999	Human tumor necrosis factor-alpha (TNF-alpha)	The transgenic mycobacterium was more effective in inducing or upregulating a range of anticancer cytokines	[87]
	Sarah L. Young et al., 2004	Human tumor necrosis factor-alpha (TNF-alpha)	Further demonstrating that the expression of mammalian cytokines significantly increased the antitumor properties	[88]
	Wen Jian et al., 2018	MAGEA3 and SSX2	Activating the immune system and enhancing anti-tumor effects	[89]
	Hyein Jeong et al., 2021	A fusion protein of human macrophage migration inhibitory factor (MIF) and interleukin 7	Exerting a strong antitumor immune response in mice model	[90]

## Data Availability

There are no supporting data associated with this article.

## Abbreviations

Bacillus Calmette-Guerin (BCG), *M.tb* (*M.tb*), Tuberculosis (TB), toll-like receptors (TLRs).
